# Insights into the ecology of early leaf-cutter bees revealed through synchrotron X-ray tomography

**DOI:** 10.1016/j.isci.2026.116813

**Published:** 2026-07-16

**Authors:** Charlie Woodrow, Robin Von Allmen, Emily Baird, Mario Vallejo-Marin

**Affiliations:** 1 Department of Ecology and Genetics, Uppsala University, Uppsala, Sweden; 2 Department of Zoology, Stockholm University, Stockholm, Sweden

**Keywords:** Acari, Apoidea, commensalism, interactions, paleontology, pollination

## Abstract

During the Paleogene, bees diversified extensively after expanding from tropical regions into northern forests surrounding the future Baltic Sea, evolving alongside diverse angiosperms. Most fossil bee diversity is preserved in amber, which captures morphology but rarely behavior or ecology. Using synchrotron X-ray imaging, we describe a new leaf-cutter bee species, †*Protolithurgus acarophorus* sp. nov., from two specimens in Baltic amber. This is only the second known record of the extinct tribe †Protolithurgiini, which displays both ancestral and derived megachilid traits. The holotype preserves exceptional anatomical detail and reveals ecological insights, including compacted floral rewards in the hind-leg scopa, a pollen-carrying strategy rare in living Megachilidae. We also document the first fossil bee-mite association, suggesting unspecialized kleptoparasitic interactions, and reconstruct thoracic musculature dimensions. Together, these findings provide rare evidence of the foraging ecology, parasite relationships, and biomechanics of the earliest leaf-cutter bees.

## Highlights

• A new species of extinct leaf-cutter bee is described from Eocene Baltic amber
• The holotype shows exceptional preservation of pollen-carrying structures
• The specimen also shows the first known fossil bee-mite associations
• Morphology of the thorax and musculature allows inferences of foraging ecology

## Graphical abstract

**Figure:**
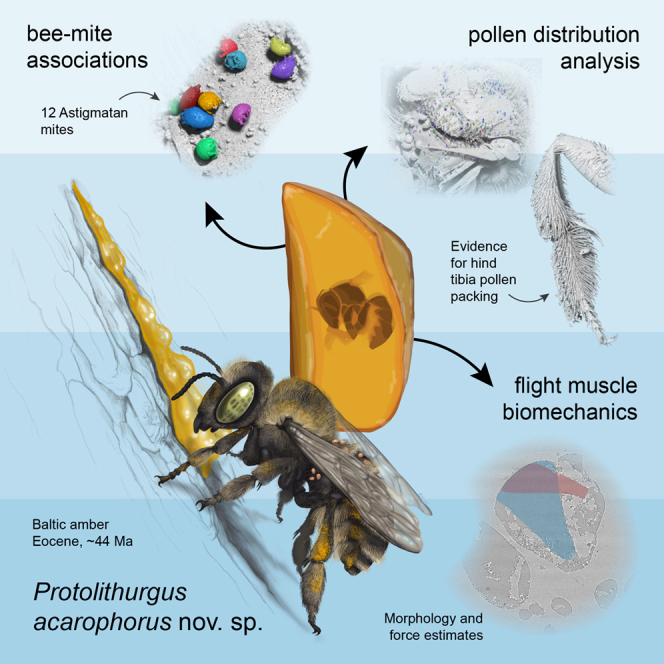

## Teaser

Zoology; Entomology; Evolutionary biology; Paleobiology

## Introduction

Leaf-cutter bees (Apoidea: Anthophila: Megachilidae) are a diverse family of more than 4,000 bee species.^1^^,^^2^^,^^3^ They are distinguished by their pollen-carrying scopa (setae or hairs) that occur on the ventral surface of the abdomen rather than on the hind legs as in many other bees^2^ and by their diverse nesting strategies, including the use of pre-existing cavities (hollow stems, wood borings, and snail shells) and the construction of brood cells out of leaves, mud, and plant resins.^2^^,^^4^^,^^5^^,^^6^ Their resource collection and nest-building behaviors also shape microhabitat communities, which encompass a broad diversity of mutualists, commensalists, predators, and parasites.^7^^,^^8^ These behaviors and interactions with other taxa are thought to have opened novel niches and contributed to their diversification.^9^ However, the behavior and ecology of early leaf-cutter bees remain poorly understood, limiting our understanding of megachilid evolutionary history.

The Megachilidae are documented through a relatively rich fossil record compared with other bee families, likely due to a bias in the fossil record toward resin-associated, forest-dwelling taxa.^3^^,^^10^ These fossils comprise many individuals from the Eocene of the Northern Hemisphere, including numerous direct preservations in fossiliferous resins^11^ and impressions of their signature bite marks in fossilized leaf tissue found globally, including in Europe, South America, and Australia.^12^^,^^13^^,^^14^ Of the Eocene amber specimens of the Baltic and Ronvo (Ukrainian) deposits, two subfamilies are currently documented: the Lithurginae and the Megachilinae. The latter subfamily includes the most species-rich megachilid genus in the fossil record, the *Ctenoplectrella*, which currently comprises six species.^15^ All the known fossil megachilids display a mosaic of traits seen across living taxa and are known from few specimens, making confident phylogenetic positioning challenging at present.^3^^,^^10^

One tribe in particular that stands out in the fossil record of the family Megachilidae is the †Protolithurgiini (Lithurginae), which comprises only a single monotypic genus known from Eocene Baltic amber, *†Protolithurgus*. The type species, †*P. ditomeus*, shows several traits unique among the extant and extinct Megachilidae.^10^ In particular, they appear to have pollen-collecting scopa on the hind legs which are absent in almost all living Megachilidae. Other fossil taxa of a similar age (†*Gyptapis*, *Glaeosmia*, and †*Ctenoplectrella*) also present hairs on the tibia with possible pollen-collecting function.^10^ They also possess dense pollen-collecting scopa the metasomal sterna, which is the plesiomorphic condition of the family. †*Protolithurgus* also has distinctive traits that characterize the extant tribe Lithurgini, but with slight differences, and has thus been placed within the Lithurginae as the sister to Lithurgini.^3^^,^^10^ This genus thus represents an important taxon within the evolutionary history of the Megachilidae and may aid our understanding of the evolution of early leaf-cutter bee morphologies, ecologies, and behaviors. However, at present, the †Protolithurgiini is represented by only a single specimen.^10^

Here, we reveal two female specimens of a newly described species of the genus *†Protolithurgus* from an inclusion in Baltic amber (Eocene, 48–34 Ma).^16^^,^^17^ The remarkable preservation of the holotype provides broad new insights into the ecology of early leaf-cutter bees. In particular, we present the distribution and description of pollen grains across the body and provide evidence of the use of the hind tibial scopa for pollen collection. Furthermore, we present the first known fossil bee-mite association, with the holotype bearing 12 astigmatan mites, mostly situated at the connection between the thorax and first abdominal segment. Finally, we reveal internal preservation of the morphology of the flight muscles of the thorax. Together, these insights from this excellently preserved specimen provide substantial new understanding of the ecology of this enigmatic tribe of extinct leaf-cutter bees.

## Results

### Systematic paleontology I: Bee

Tribe PROTOLITHURGINI Engel 2001

Genus *†Protolithurgus* Engel 2001.

Type Species: *†Protolithurgus ditomeus* Engel 2001.

***†****Protolithurgus acarophorus*, sp. nov.

**LSID** urn:lsid:zoobank.org:act:47103BD0-AB97-401A-8D77-CD808B8B8617 (Figures 1, 2, 3, 4, and S1–S6).

**Figure 1:**
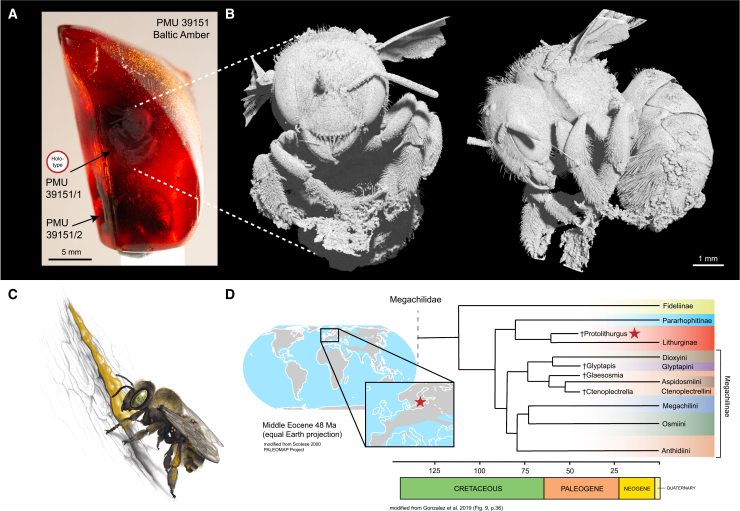
*†Protolithurgus acarophorus* sp. nov (A) Amber stone with position of holotype 39151/1 and paratype 39151/2 with superimposed image of 39151/1 to aid in visual orientation. (B) Ventral and lateral habitus of *†P. acarophorus* 39151/1 from synchrotron μ-CT data. (C) Illustrated reconstruction of the species (illustration by Denitsa Peneva). (D) Geographical origin of the specimen from a projection of the middle Eocene and phylogenetic position of the genus *†Protolithurgus*; middle Eocene projection is modified from PALEOMAP project (Scotese, 2000)^18^; gray areas indicate the land masses 48 Ma, while the white lines represent modern geography. Phylogeny simplified and redrawn from Gonzalez et al.^19^ Note that although the PALEOMAP projection is dated to 48 Ma; Baltic amber is dated to anywhere in the range of 48–34 Ma. Scale bars in (A), 5 mm. Scale bars in (B), 1 mm.

**Figure 2:**
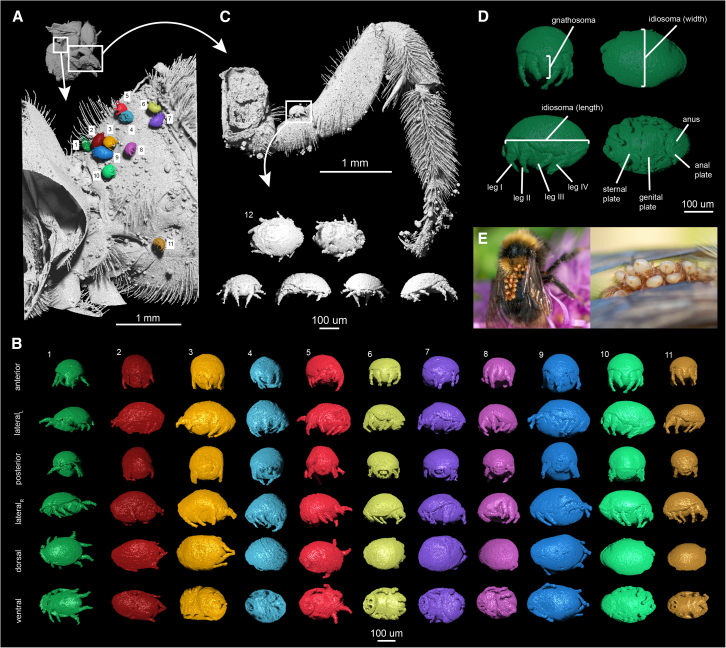
Kleptoparasitic astigmatan mites of *†P. acarophorus* sp. nov (A) Distribution of mites on the posterior surface of thorax (propodeum). (B) Detailed morphology of each mite in anterior, lateral_L_ (left), posterior, lateral_R_ (right), dorsal, and ventral orientations. (C) Additional twelfth mite on the left mesotrochanter and different orientations. (D) Mite anatomy and measured features. (E) Presence of extant bee-mites on a *Bombus* sp., highlighting the common parasite load of bees (note these are not of the cohort Astigmata but shown to indicate the common presence of high parasite loads in extant bee taxa). Scale bars in (A), 1 mm. Scale bars in (B), 0.1 mm. Scale bars in (C), 1 mm (top) and 0.1 mm (bottom). Scale bars in (D), 0.1 mm.

**Figure 3:**
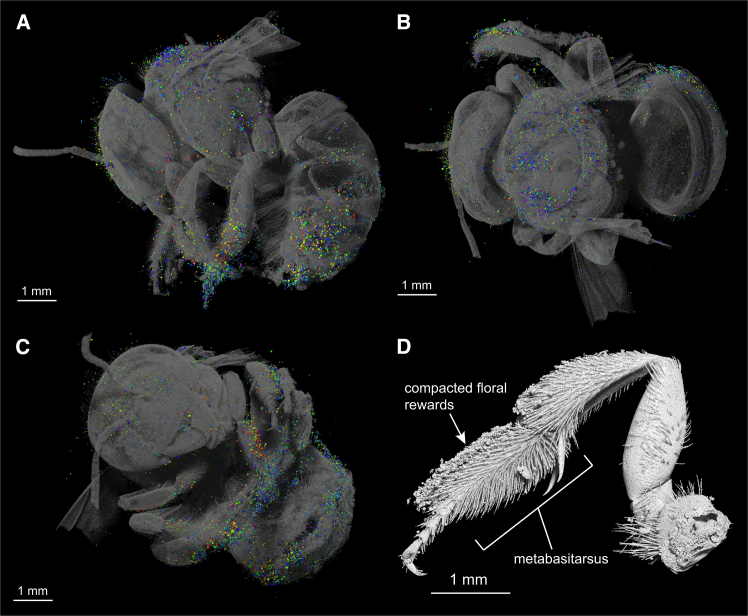
Pollen distribution in the holotype of *†P. acarophorus* sp. nov (A) Lateral view. (B) Dorsal view. (C) Ventral view. (D) Compacted floral rewards in the scopa of the metabasitarsus (hairs of the hind legs). All scale bars, 1 mm.

**Figure 4:**
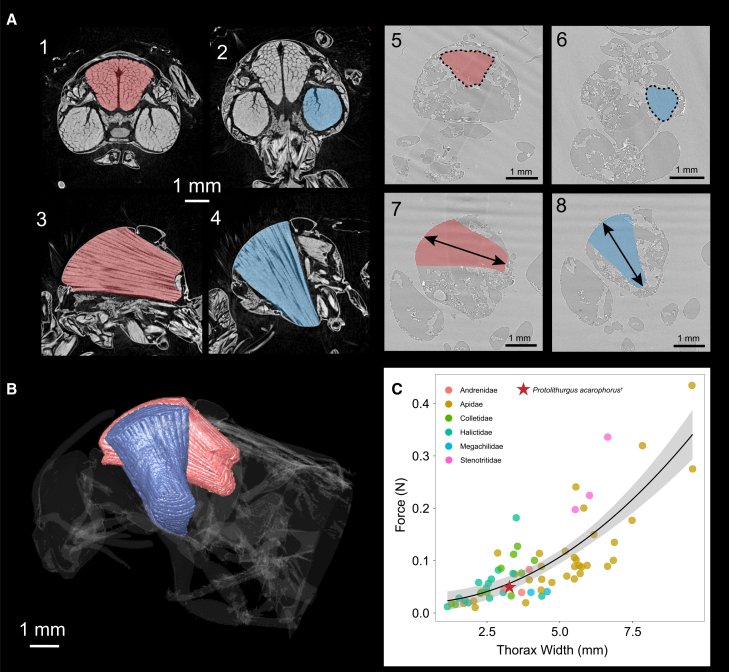
Thoracic muscle anatomy and biomechanics of *†P. acarophorus* sp. nov (A) Thoracic musculature of the buff-tailed bumblebee *Bombus terrestris* compared with *†P. acarophorus*, highlighting the cross-sectional areas of the dorso-longitudinal muscles (1,5), cross-sectional areas of the dorso-ventral muscles (2,6), fiber lengths of the dorso-longitudinal muscles (3,7), and fiber lengths of the dorso-ventral muscles (4,8). (B) 3D reconstruction of the muscles of *B. terrestris.* (C) Relationship between thorax width and peak force of the thoracic muscles measured across extant bee families. Line and shaded area represent mean and standard error, respectively. Data are from Vallejo-Marin et al.^20^ All scale bars, 1 mm.

#### Diagnosis

Short and flat first metasomal tergum (T1). Third labial palpus on the same axis as the second. Metabasitibial plate present. Separated easily from type species *P. ditomeus* based on clypeus, which is smooth, not protuberant in lateral view, extending to or slightly below the lower tangent of compound eyes, and with crenate lower margin rather than a smooth margin. Mandible bidentate as in *P. ditomeus* but with a longer outer ridge and well-defined condylar ridge. It also differs in body dimensions (see following section).

#### Description

Preserved body length 10.30 mm (head to basal portion of T5; Figure 1). Head ∼1.28 times wider than long (length 2.66 mm, width 3.40 mm; Figure S1). Clypeus smooth, extending to or slightly below the lower tangent of compound eyes; crenate lower margin (Figure S1). Upper interorbital distance 2.14 mm; lower interorbital distance 1.96 mm. Interocellar distance 0.36 mm; ocellocular distance 0.65 mm; median to lateral ocellus 0.09 mm; ocelloccipital distance 0.70 mm (Figure S1). Intertegular distance 2.96 mm, thorax width 3.56 mm (Figure S4). Epistomal suture forming an obtuse angle; supraclypeal area smooth, continuous with level of clypeus (Figure S4); subantennal sutures meeting outer margin of antennal sockets (Figure S1); flagellomere F1 distinctly longer than F2 (Figure S1). Compound eyes emarginate (Figure S1). Preoccipital ridge rounded. Glossa 1.63 mm long with ∼90 annular segments and flabellum present, rounded (Figure S1); third labial palpus on same axis as second (Figure S2); galea flattened, length 1.25 mm (Figure S2). Tibiae not spiculate; metabasitibial plate present, imbricate, large and broadly rounded (Figure S3); claws cleft (Figure S3); arolium present (Figure S3). Metasoma with sternal scopa; T1 short (0.57 × T2 length), flat in lateral view (Figure S5). Lancet of stinger 1.5 mm (Figure S5). Scutellum slightly more than three times length of metanotum; metanotum about as long as basal area of propodeum (Figure 4). Basal vein straight; first submarginal cell apparently equal in length to second submarginal cell; other wing components not identifiable. Pygidial plate absent (Figure S5). Labrum, clypeus, supraclypeal area, face, vertex, gena, and postgena imbricate. Mesosoma imbricate. Metasoma imbricate. Clypeus, supraclypeal area, and face below level of antennal sockets with scattered, simple, erect, long (2–3× ocellar diameter [OD]) setae; clypeal margin with patches of setae between crenation. Postgena with long (2–3.5× OD), erect, simple setae. Pronotum with minute, sparse, simple setae except dorsolateral angle toward pronotal lobe with longer, sparse (1× OD) setae. Mesoscutum with erect, sparse, simple setae. Scutellum with erect, plumose (1–2× OD) setae. Metanotum without setae. Metepisternum and lateral surface of propodeum with sparse, suberect, simple (1× OD) setae; pre-episternum and mesepisternum with scattered, long (2–3× OD) setae. Basal area of propodeum without pubescence. Metafemur without scopal setae; metatibia and metabasitarsus with dense, scopal setae, longer on metabasitarsus (Figure S3). Metasomal terga with sparse, simple setae; metasomal sterna with dense, elongate (4–5× OD, max length 1.62 mm), simple, scopal setae (Figure S5).

#### Material

Two specimens in single Baltic amber stone, PMU 39151 (PMU; Palaeontological collections, Museum of Evolution, Uppsala University, Sweden) from the Palmgren amber collection. John Palmgren, a Uppsala geologist, bought the collection in 1936 from a Danish collector named F. Enstedt at Hou in Ulsted, Jylland, on behalf of Professor Carl Wiman at the museum in Uppsala. Precise origin of stone within Baltic states is unknown. *Holotype*. Female, PMU 39151/1. Located in the middle of stone. Challenging to see with the eye due to the dark, opaque color of stone. Full specimen intact, but the apex of wings is missing from current scan data. *Paratype*. Female, PMU 39151/2. Located at top of stone. Challenging to see with the eye due to the dark, opaque color of stone. Badly damaged from drilling of hole to form pendant (Figure S6). Wings likely polished off the edge of stone during preparation. Mid-legs well preserved. Specimens misdiagnosed as “*Ctenoplectrella* sp.” so may occur under this synonym in specimen records.

#### Etymology

Derived from the combining form acaro- (from Greek akari, “mite”) and -phorus (from Greek phoros, “bearing”), meaning “mite-bearing,” due to the presence of numerous mites on the thorax (see comments).

#### Comments

Placed within *†Protolithurgus* based on short and flat first metasomal tergum (T1), third labial palpus on the same axis as the second, and presence of metabasitibial plate (see Diagnosis for comparisons within genus). The holotype of *†Protolithurgus acarophorus* has eleven astigmatan mites attached to the posterior margin of the thorax and metasomal tergum (Figures 2, S3, and S4) as known from some modern Megachilidae.^21^ There is also an additional twelfth mite on the left mesotrochanter (Figures 2 and S3). Prior to this specimen, the only direct fossil evidence of mite-associated Hymenoptera was on the dolichoderine ant *Ctenobethylus goepperti*,^22^ making this the first direct evidence of bee-mite associations in the fossil record.

### Systematic paleontology II: Mites

Cohort ASTIGMATA Canestrini, 1891.

Family indet.

(Figures 2, S3, and S4).

#### Material

Syninclusion of the holotype of *†Protolithurgus acarophorus* (Apoidea: Megachilidae: †Protolithurgiini) from Eocene Baltic amber (PMU 39151; Palaeontological collections, Museum of Evolution, Uppsala University, Sweden). 12 specimens in association with a single solitary bee as their host (Figure 2), with 11 on propodeum (Figures 2A and 2B) and 1 on left mesotrochanter (Figure 2C).

#### Description

Phoretic deutonymphs of Astigmata; dorsal idiosoma convex, rounded to oval in dorsal view, length 244.6 ± 27.2 μm, width 177.3 ± 16.5 μm (*N* = 12; Figure 2D; Table S1); holodorsal shield present, surface smooth, more detail not resolvable. Anal plate visible, clear attachment function. All legs short, maximum leg length 113.4 ± 11.5 μm (*N* = 12; Figure 2D and Table S1), legs IV not notably shorter than other legs, and lacking modification as in extant Astigmata. Setae and fine features not resolvable. Gnathosoma length 59.4 ± 6.5 μm (*N* = 12; Figure 2D; Table S1).

#### Comments

The unmodified legs IV argue strongly that this specimen does not belong to *Vidia*, a mite genus common on extant Megachilidae.^21^ The presence of unmodified legs IV is a plesiomorphic character state, which makes this fossil particularly interesting for understanding the evolution of bee-associated mites, as only a few extant mite families retain this feature (Jason Dunlop and Pavel Klimov, personal communication). Unfortunately, as the amber is opaque and the mites were only identified after imaging, we do not have higher resolution images of these mites; however, additional synchrotron scanning or high-resolution confocal^23^ will allow for higher level taxonomic identification in the future.

### Early Megachilidae used hind leg scopa to carry pollen from diverse plants

Both the holotype and paratype of *†P. acarophorus* showed significant amounts of external particulate matter, which we interpret as pollen grains on the basis of their location in the pollen-collecting appendages and setae, their pollen-like morphology, and the presence of shrunken internal organic matter, most likely the exine, consistent with pollen inclusions in amber (Figure S7). In the holotype, these potential pollen grains were present on all major body segments, with their distribution reflecting the spaces that extant bees struggle to groom, including the dorsal thorax, abdomen, face, and spaces between body segments (Figure 3).^24^^,^^25^ We identified and measured 29,579 potential pollen grains, which were filtered based on the size and aspect ratios of known pollen grains from Eocene amber^26^ to produce a selection of 14,875 pollen grains, of which 9.78% (1,365) were found in association with the head, 23.74% (3,531) with the thorax, 27.95% (4,157) with the abdomen, and 39.14% (5,822) with the legs. These palynomorphs (pollen-like objects) had a distribution of spherical diameters which showed two clear peaks in abundance, one at ∼16 μm, another at ∼31 μm (Figure S7), which could correspond to pollen of the Fagaceae and Betulaceae based on known pollen from Eocene amber,^26^ although it should be noted that size is not sufficient for the identification of pollen. For larger pollen grains (i.e., larger than 75 μm) with characteristic morphologies such as the presence of sacci (Figure S7), we were able to reach higher level identification using manual segmentation. This revealed the presence of pine (Pinaceae: *Pinus*; Figure S7), cedar (Pinaceae: *Cedrus*; Figure S7), and several unidentifiable tricolpate forms^26^^,^^27^; however, these larger pollen-like structures were low in abundance, indicating they were likely collected through exposure in the environment rather than through direct foraging. The holotype also has a large region of compacted structures on the hind legs, positioned in the elongated setae of the metabasitarsus (Figure 3D). Their position indicates strongly that these are compacted floral rewards and that the setae have a scopal function.

### Flight anatomy and function can be extracted from fossil geometries

In addition to the excellently preserved external features, the internal anatomy hints at the morphology of other features within the holotype of *†P. acarophorus*. In particular, the internal structure has been preserved in a way that has retained the gross morphology of the indirect flight muscles of the thorax (Figure 4). This allows us to estimate some properties of the internal muscle morphology and physiology of this species. For example, the maximum fiber length of the dorso-longitudinal muscles (DLMs) and dorsoventral muscles (DVMs) was estimated to be 2.77 and 2.46 mm, respectively. We can also estimate the physiological cross-sectional area (PCSA) of the DLMs and the left DVM, which were found to be 1.24 and 0.76 mm^2^, respectively. Based on this value, we can also calculate the maximum force of the flight muscles by multiplying the PCSA of the DLMs by the tetanic stress of bee flight muscle (∼40 kN/m^2^),^28^ which yields a force estimate of 0.0496 N. For the DVMs, if we assume perfect symmetry (and thus the combined PCSA of left and right DVMs is 0.152 mm^2^), we obtain a force estimate of 0.0608 N. These estimates fit well within the expected force for a bee of this size (Figure 4C), aligning closely with the value obtained if we predict force from thorax width (3.56 mm) using phylogenetically controlled models (0.0605 N).^20^ Similar published models can also be used to estimate a peak thorax acceleration of ∼64.8 m s^−2^.^20^ We can also use measurements of thorax width and body length to predict flight-based foraging distances based on established models.^29^^,^^30^ Based on an inter-tegular distance of 2.96 mm, the typical homing distance of *†P. acarophorus* can be estimated as 0.89 km.^30^ Furthermore, based on a body length of 10.3 mm, we obtain a foraging distance of 0.33 km.^29^ However, such predictions on foraging distance should be used with caution, as they assume that scaling relationships have remained conserved over millions of years, and do not account for environmental parameters, such as temperature, which may affect foraging range.

## Discussion

### Pollination ecology of early Megachilidae

The fossil record indicates that the Paleogene is the most diverse period for extinct bees; however, this observation is likely heavily biased by forest dwelling and resin-collecting bees, which are more likely to be captured in tree resins.^10^^,^^13^ The specimen studied here was historically identified as a species within the genus *Ctenoplectrella* Cockerell (Megachilinae: Ctenoplectrellini), but our μCT data revealed the morphology of this specimen in fine detail and allowed us to identify this as a new species in the unique genus *†Protolithurgus* Engel 2001 (Megachilinae: †Protolithurgiini), making *†P. acarophorus* only the second described species in this tribe.^10^ The †Protolithurgiini are unique among the living and extinct leaf-cutter bees, being both outside of the largest subfamily Megachilinae (Figure 1),^3^ and displaying a unique combination of ancestral and derived leaf-cutter bee traits.^10^ Namely, they have a short and flat first metasomal tergum, otherwise only found in extant Lithurgiini, and are thought to have pollen-collecting scopa on both the hind tibiae and the metasomal sterna.^3^^,^^10^ Here, we provide strong evidence for the latter assumption, by revealing a concentration of spherical and broken structures in the setae of both metabasitarsi (Figure 3). Given their presence in both hind legs, the abundance of pollen across the body, and a lack of similar structures in the scan data, these are very likely compacted floral rewards.

The evolution of pollen-collecting structures (scopa) in Megachilidae represents a key aspect of morphological and behavioral diversification within the family. While we here provide evidence of hind tibial scopae in †Protolithurgiini, other fossil megachilids including *Gyptapis*, *Glaeosmia*, and *Ctenoplectrella* show similar structures, but a pollen collection function has not been confirmed.^10^ Such features are not only restricted to this extinct tribe but also retained in several early-diverging extant groups such as *Pararhophites*, *Apidosmia*, Fideliinae, and Neofideliinae.^3^^,^^31^^,^^32^^,^^33^ In Fideliinae and Neofideliinae, these scopa are likely to function for nest excavation rather than pollen collection,^2^ meaning the function of scopa in extinct Megachilidae has remained uncertain. We demonstrate that in *†P. acarophorus*, these scopae were likely used for pollen collection. As well as scopae on all legs (including the hind tibiae), these living and extinct species also have pollen collecting hairs on the ventral metasoma, indicating a plesiomorphic condition involving multiple pollen-carrying surfaces. This broader distribution of scopal structures has been documented in taxonomic and phylogenetic treatments of these groups.^3^^,^^31^^,^^32^ The reduction or complete loss of the hind tibial scopa in most Megachilidae should thus be interpreted as a derived condition,^2^ as concluded from morphological studies.^3^ Comparative morphological and phylogenetic studies further suggest that scopal redistribution, from a generalized condition involving multiple appendages (e.g., legs and metasoma) to a more specialized abdominal setae, occurred in conjunction with other key innovations, including modifications of mandibular structures and nesting behaviors.^3^ Furthermore, Megachilidae specialized on composite flowers (Asteraceae) tap their abdomens to collect pollen,^34^ indicating modifications in foraging behavior with scopal redistribution. This specimen thus provides direct evidence that extinct Megachilidae possessed multiple pollen-carrying surfaces, including those known from wider bee taxa, and this was later reduced to primary use of abdominal setae in most lineages.^3^ The general distribution of pollen across the body aligns with the regions where bees typically struggle to reach during grooming,^25^ namely the dorsal abdomen and the connection between thorax and abdomen (Figure 3). Selection for specific pollen placement may have shaped scopal redistribution in Megachiliae.

When bees collect and compact floral rewards, pollen grains are tightly associated and often damaged during packing,^35^ making separation of individual pollen grains within the metabasitarsi challenging. However, in other parts of the body, the pollen grains remain intact, allowing for some degree of identification and distribution analysis. In fossil palynology—the study of fossilized microscopic particulate matter such as pollen, spores, and other palynomorphs—species-level identification of even fully intact pollen grains is often unfeasible without the preservation of the original source (flower, fungus, etc.).^26^ Instead, palynomorphs are identified by morphotypes, categorized based on size, shape, surface structure, and abundance, and then assigned by affinity to the most likely family.^26^ Only in cases of unique morphologies is it possible to confidently assign lower taxonomic ranks. In our scan, we were able to clearly identify pollen grains from coniferous gymnosperms, due to their air sacs, or sacci, that help the pollen grains carry in the wind.^27^^,^^36^ This pollen can be distinguished as a mix of pine (Pinaceae: *Pinus*; Figure S7) and cedar (Pinaceae: *Cedrus*; Figure S7) based on morphological proportions of the pollen grain and sacci.^26^^,^^27^ This pollen almost certainly ended up in the setae of the body by chance and was not actively foraged, but the presence of Pinaceae pollen, in addition to the fact that the specimen is preserved as an inclusion in amber, strongly indicates that *†P. acarophorus* was foraging for coniferous tree resins for nest construction as is known from extant Megachilidae.^2^^,^^4^^,^^5^^,^^6^ This insight also favors a Pinaceae, rather than Sciadopityaceae (umbrella pine), botanical origin of Baltic amber.^10^^,^^37^^,^^38^ As for angiosperm pollen identification, our confidence is low due to the fact that even at 2.6 μm, our 3D pixel resolution is too poor to distinguish the smaller pollen grains as one can do through confocal microscopy or by dissolving the amber stone.^23^^,^^26^ It is also possible that some of the palynomorphs identified here do not represent pollen grains but fungal spores, which are equally abundant and incredibly diverse in Eocene amber.^26^ However, we can confidently identify these structures as palynomorphs and not mineral inclusions due to the shrunken organic matter, most probably the pollen exine or perisporidium, which remains intact inside the space the hydrated pollen grain would have occupied as the amber was forming (Figure S7).^26^ Considering these limitations, we can only predict that this likely comes from plant families already known from Baltic amber; Betulaceae (birch), Fagaceae (beeches, chestnuts, oaks), Hamamelidaceae (witch-hazel), and Juglandaceae (walnut).^26^ Given our predicted estimates of foraging range in *†P. acarophorus* of 0.33–0.89 km, it is likely that the coniferous forests of the Eocene were diverse in local plant species composition, but such predictions should be used with caution as they assume that body size and foraging range scaling relationships have remained conserved through time and across taxa.

While our identification of angiosperm pollen is limited, our understanding of the pollen load and landscape of *†P. acarophorus* could be further investigated. Across the body, we identified 14,875 likely pollen grains (Figure 3). This is a conservative estimate, as it does not account for the compacted pollen in the hind legs which is not possible to isolate. This pollen load fits within the broad range of pollen loads measured from freely foraging bees, whereby the number of pollen grains can range from ∼5,000 to over 100,000.^39^^,^^40^ Furthermore, the distribution of pollen across the body was similar to that described for freely foraging bumblebees and honeybees, whereby we found 9.78% of pollen in association with the head (*Bombus* average 10.01% and *Apis* average 12.12%),^39^^,^^40^ 23.74% with the thorax (*Bombus* average 22.04% and *Apis* average 23.62%),^39^^,^^40^ 27.95% with the abdomen (*Bombus* average 26.25% and *Apis* average 9.36%),^39^^,^^40^ and 39.14% with the legs (*Bombus* average 33.3% and *Apis* average 45.33%).^39^^,^^40^ These pollen grains were mostly small in size, with size distribution histograms showing two clear peaks at diameters of ∼16 and ∼31 μm (Figure S7). which could correspond to pollen of the Fagaceae and Betulaceae.^26^ Larger pollen grains, such as those of the aforementioned gymnosperms, were low in abundance, and thus likely only collected through passive exposure in the environment rather than active foraging. Further work comparing the pollen landscapes of extant bee species, combined with higher resolution imaging for further identification of the pollen in this fossil, would be useful for understanding the evolution of pollen placement strategies of angiosperms, and resource collection strategies of the Megachilidae. Further higher resolution imaging of *†P. acarophorus* may currently be beyond technical limits without potentially invasive cutting and polishing of the amber stone but, if possible, could greatly enhance our understanding of early leaf-cutter bee foraging ecology.

### Parasite networks of the Eocene

In addition to providing insights into the foraging ecology of extinct leaf-cutter bees, the holotype of *†P. acarophorus* also offers a rare glimpse into the parasite networks of the Eocene, with 11 mites present on the posterior margin of the thorax and metasomal tergum, and a further twelfth individual on the left mesotrochanter (Figure 2). Being found in association with a solitary bee species indicates that these are most likely kleptoparasitic astigmatan mites (Acari: Parasitiformes), a diverse group with around 5,000 extant species.^41^ This is further confirmed by the presence of a ventral attachment organ (Figure 2D), indicating that these specimens are phoretic deuteronymphs of Astigmata, using *†P. acarophorus* to move between resources before a free-living adult phase consuming fungi and/or plant resources.^21^^,^^42^ Extant members of the cohort Astigmata include economic pests of food and stored products, as well as kleptoparasites of many birds and mammals.^21^

While the Astigmata are greatly understudied, some affinities with the Megachilidae are known today. Perhaps the largest group is of the genus *Vidia* (Ensliniellinae), which are phoretically associated with megachilids of the genera *Anthidium*, *Creightonella*, and *Megachile*.^42^ These are free-living mites feeding on fungi on leaves within the bee’s nest, and the deutonymphs attach to pupae prior to eclosion to move to the next nest.^42^ If a similar behavior was utilized by these mites to transport between resources, it could support our hypothesis of fungal spores amongst pollen grains and other palynomorphs. Interestingly, while most extant bee mites of this group display modified legs IV, allowing them to hang onto the hair of their host,^43^ the Astigmata in association with *†P. acarophorus* lack this modification, indicating reduced specialization to its host. Looking to the extant Lithurginae, the closest relatives of *†P. acarophorus*, there is good documentation of astigmatan mites of the family Chaetodactylidae whose hosts include bees of the genera *Lithurgus*, *Trichothurgus*, and *Microthurge*.^43^ This family could thus be a good candidate for higher level taxonomic identification, but improved imaging resolution is needed.

More generally across the Eocene amber fossil record, the presence of mites and their hosts together is sparse but diverse. Where examples have been found, most are associations with Coleoptera, including longhorn beetles (Cerambycidae),^44^ cryptophagids (Cryptophagidae),^45^ and bark beetles (Trogossitidae).^46^ These associations likely arise from communal living in and around tree barks, and are certainly most common due to their close affinity with tree resins.^16^^,^^47^ Other syninclusions of mites and their hosts are known from spiders (Araneae: Dysderidae),^48^ whip scorpions (Arachnida: Thelyphonida),^49^ bristly millipedes (Polyxenida: Polyxeninae),^50^ and termites (Blattodea: Isoptera).^51^ In the Hymenoptera, the only fossil record of a mite with its host represents an association between a Mesostigmatid mite of the genus *Myrmozercon* on the ant *Ctenobethylus goepperti* (Hymenoptera: Formicidae: Dolichoderinae),^22^ an interaction also described from Baltic amber. Thus, the holotype of *†P. acarophorus* and its phoretic astigmatan passengers represent the first definitive association between bees and mites in the fossil record. While the Astigmata may represent a distant sister group to the economically important bee mite *Varroa destructor*,^52^ we now know that the widespread interaction network between bees and mites dates back at least 44 million years.

### Flight muscle biomechanics

In the holotype of *†P. acarophorus*, we see remnants of the morphology of the thoracic musculature within the thorax.^52^^,^^53^^,^^54^ This has not occurred through preservation of the muscle tissue itself, but in partial preservation of the space the muscles did not occupy (Figure 4). Using this information, we were able to obtain several traits of the flight muscle morphology and biomechanics. Firstly, we were able to obtain the maximum and minimum fiber lengths of the DLMs and DVMs from the overall geometry of muscles combined with the morphology of the thorax (Figure 4). We were also able to extract an estimated PCSA of the muscles to calculate a peak force range of 0.0496–0.0608 N. These values are very well aligned with our peak force estimate based on thorax width alone (0.0605 N; Figure 4D). Based on the width of the thorax, we also estimated a peak thorax amplitude during flight muscle operation of ∼65 m s^−2^.^20^ This is on the low end of thorax amplitudes which simply reflects the small size of *†P. acarophorus* compared with extant bee taxa which have amplitudes ranging from ∼2 to 1500 m s^−2^.^20^ In theory, this information could be used to help us understand the evolutionary biomechanics of extinct bee flight muscles, but for now we lack the morphological data for extant species which would be needed to fully utilize this information. However, we highlight that the holotype of *†P. acarophorus* may be a useful specimen to understand the evolution of bee flight musculature in the future, and for inferring the evolution of other flight muscle functions such as defense, communication,^20^ and buzz pollination.^53^

The Paleogene is known for its diversity of fossil bees.^10^ Here, we expanded this diversity through the description of a new species of the elusive †Protolithurgiini. Our ecological insights shed new light on the pollen-collecting strategies, parasite interactions, and morphology and biomechanics of early leaf-cutter bees. The pollen identified on the body of *†P. acarophorus* also strengthens our current understanding of the rich temperature-subtropical forests this bee would have occupied almost 50 million years ago,^26^^,^^54^ which included a mix of pines and cedar, which early Megachilids were using for resin collection for nest construction. We highlight that such insights are made possible through high-resolution tomography, and imaging of other amber bee inclusions, combined with much-needed 3D morphological data from extant species, have the capacity to transform our understanding of extinct bee ecologies.

### Limitations of the study

Our description of *†P. acarophorus* and its associated ecological insights provide a deeper understanding of Megachilidae biology, elucidating in particular the pollen-carrying role of the hind-leg scopa. However, at this stage, we are limited in a deeper understanding of these ecological insights, mostly due to technological limitations. Further higher resolution imaging of the identified mites and pollen could provide better confidence in identification. Furthermore, while we provide predictions of flight ecology and biomechanics from measurements of the holotype, such predictions assume that the scaling of body size with the predicted parameters has remained constant over millions of years, which requires further investigation.

## Resource availability

### Lead contact

Further information and requests for resources should be directed to the lead contact, Charlie Woodrow (charlie.woodrow@gmail.com).

### Materials availability

The specimen described in the study is deposited in the paleontological collections, Museum of Evolution, Uppsala University, Sweden, under the specimen code PMU 39151. This study did not use or generate any existing or new reagents.

### Data and code availability

• All key data are reported within the paper, see key resources table.
• This paper does not report original code.
• Any additional information required to reanalyze the data reported in this paper is available from the lead contact upon request.

## Acknowledgments

We thank Jan Ove R. Ebbestad, Senior Curator of the paleontological collections at the Uppsala Museum of Evolution, for providing specimen information and arranging loans. We also appreciate the support of beamline scientists Shashidhara Marathe and Kaz Wanelik for aiding in synchrotron data processing. We thank Fernando Montealegre-Z for allowing us to use the μ-CT scanner at the 10.13039/100032400University of Lincoln for preliminary imaging. We also thank Victor Hugo Gonzalez for discussions of scopa evolution in the Megachilidae and Jason Dunlop and Pavel Klimov for guidance on mite identification. Thanks to Per Ahlberg for initial discussions regarding synchrotron fossil data and to Sifra Bijl for organizing access to tomographic data processing computers. We also thank Denitsa Peneva for bringing this specimen back to life with their beautiful illustration in Figure 1. This project was funded by a 10.13039/100011889Diamond Light Source grant awarded to C.W., E.B., and M.V.M. (project no. MG37346). C.W. and M.V.M. are also supported by a Human Frontiers Science Program grant (RGP0043/2022; “HFSP.RGP00432022.pc.gr.153603”: https://doi.org/10.52044/HFSP.RGP00432022.pc.gr.153603) awarded to M.V.M.

## Author contributions

Conceptualization, C.W.; data curation, C.W. and R.V.A.; formal analysis, C.W. and R.V.A.; funding acquisition: C.W., E.B., and M.V.M.; investigation, C.W., R.V.A., and M.V.M.; methodology, C.W., R.V.A., E.B., and M.V.M.; project administration, C.W., E.B., and M.V.M.; resources, C.W., E.B., and M.V.M.; supervision, C.W. and M.V.M.; validation, C.W., R.V.A., E.B., and M.V.M.; visualization, C.W.; writing – original draft, C.W.; and writing – review and editing, C.W., R.V.A., E.B., and M.V.M.

## Declaration of interests

The authors declare no competing interests.

## STAR★Methods

### Key resources table

**Table:** 

REAGENT or RESOURCE	SOURCE	IDENTIFIER
**Deposited data**		

3D micro-CT scan of holotype of Protolithurgus acarophorus. Available to freely download from the MorphoSource data respository	MorphoSource	Morphource: 000848144. Protolithurgus Acarophorus Holotype Full Body Ct Scan

**Software and algorithms**		

Dragonfly 3D world image analysis software	Dragonfly 3D World (2026)^55^	N/A
Fiji	Schindelin, J. et al. (2012)^56^	N/A

### Method details

#### Synchrotron μCT imaging

X-ray micro-computed tomography (μCT or SR- μCT) imaging was conducted at the I13-2 beamline at Diamond Light Source, Oxford, UK (beamtime MG37346). The holotype and paratype were imaged separately. The holotype was scanned in 2 parts to ensure the head, thorax, and abdomen were included within the image. We used a pco.edge 5.5 detector fitted with a 1.25× objective for a total magnification of 2.5× and an effective voxel size of 2.6 μm. The holotype was aligned off-centre for 360° rotation while the paratype was aligned centrally and imaged with 180° rotation. For all scans, exposure time was set to 300 ms, and a 0.072° rotation step. Projections were reconstructed into orthogonal image slices on site using in-house code. Orthogonal image slices were exported in TIFF format.

#### Segmentation and visualization

Segmentation and visualization of tomographic data was conducted in Dragonfly 3D World version 2025.1.^55^ To de-noise the data, anisotropic diffusion (with default parameters) was applied to the tomographic data after import into Dragonfly using the image filtering open plugin. Segmentation was done semi-manually, initially using the manual greyscale histogram to select the negative space of the fossil which represents the bee body. This was then refined with manual segmentation on a slice-by-slice basis to repair holes and remove artifacts following the geometry of each image slice. Morphological measurements were taken from exported orthographic projections perpendicular to the body part to be measured using Fiji.^56^

#### Pollen segmentation and measurement

To identify and measure the palynomorphs across the body of *†P. acarophorus*, we used two methodologies. First, we used a manual region of interest (ROI) analysis to manually select distinct morphologies for further investigation. Secondly, we used a multi-ROI analysis to select all possible pollen grains across the body and measure their distributions and sizes. All analyses were conducted in Dragonfly 3D World version 2025.1.^55^ In the multi-ROI analysis after objects were initially identified as potential pollen grains, we filtered these objects for equivalent spherical diameters of 10–160 μm, and aspect ratios of between >1 and 3, matching the known dimensions of pollen palynomorphs in Eocene amber.^26^ Note that the aspect ratio filtering must be set to >1, because artifacts of this workflow can render as small perfect spheres with an aspect ratio of exactly 1. Finally, we used the 3D selection tool to isolate palynomorphs associated with the head, thorax, abdomen, and legs separately, and made counts of the number of likely pollen grains within each body section.

#### Thorax force and flight distance estimates

Finally, in line with our thorax morphology and muscle morphology description, we used the thorax width of *†P. acarophorus* to predict peak force and peak amplitude (acceleration) based on MCMCglmm models from extant bee taxa accounting for phylogeny.^20^ We predicted peak force from thorax width using the model Ln(peak force) = 1.2361∗ln(thorax width)-2.1531. We also predicted peak amplitude acceleration using the model ln(peak amplitude acceleration) = 1.213∗ln(thorax width)+2.738.^20^ Estimates of homing and foraging distance follow published linear models of ln(homing distance) = −1.363 + 3.366∗ln(inter-tegular distance), and *foraging distance* = −232.28 + 54.69∗body length, respectively.^29^^,^^30^

### Quantification and statistical analysis

There are no quantification or statistical analyses to include in this study.
