# Supplementary material for: Insights into the ecology of early leaf-cutter bees revealed through synchrotron X-ray tomography
Source: iScience. 2026 Jul 16;29(8):116813. doi: 10.1016/j.isci.2026.116813 (PMC13400859; doi:10.1016/j.isci.2026.116813)
Supplement: Document S1. Figures S1–S7 and Table S1 [file mmc1.pdf]

iScience, Volume *29*

Supplemental information

Insights into the ecology of early leaf-cutter bees revealed through synchrotron X-ray tomography

Charlie Woodrow, Robin Von Allmen, Emily Baird, and Mario Vallejo-Marin

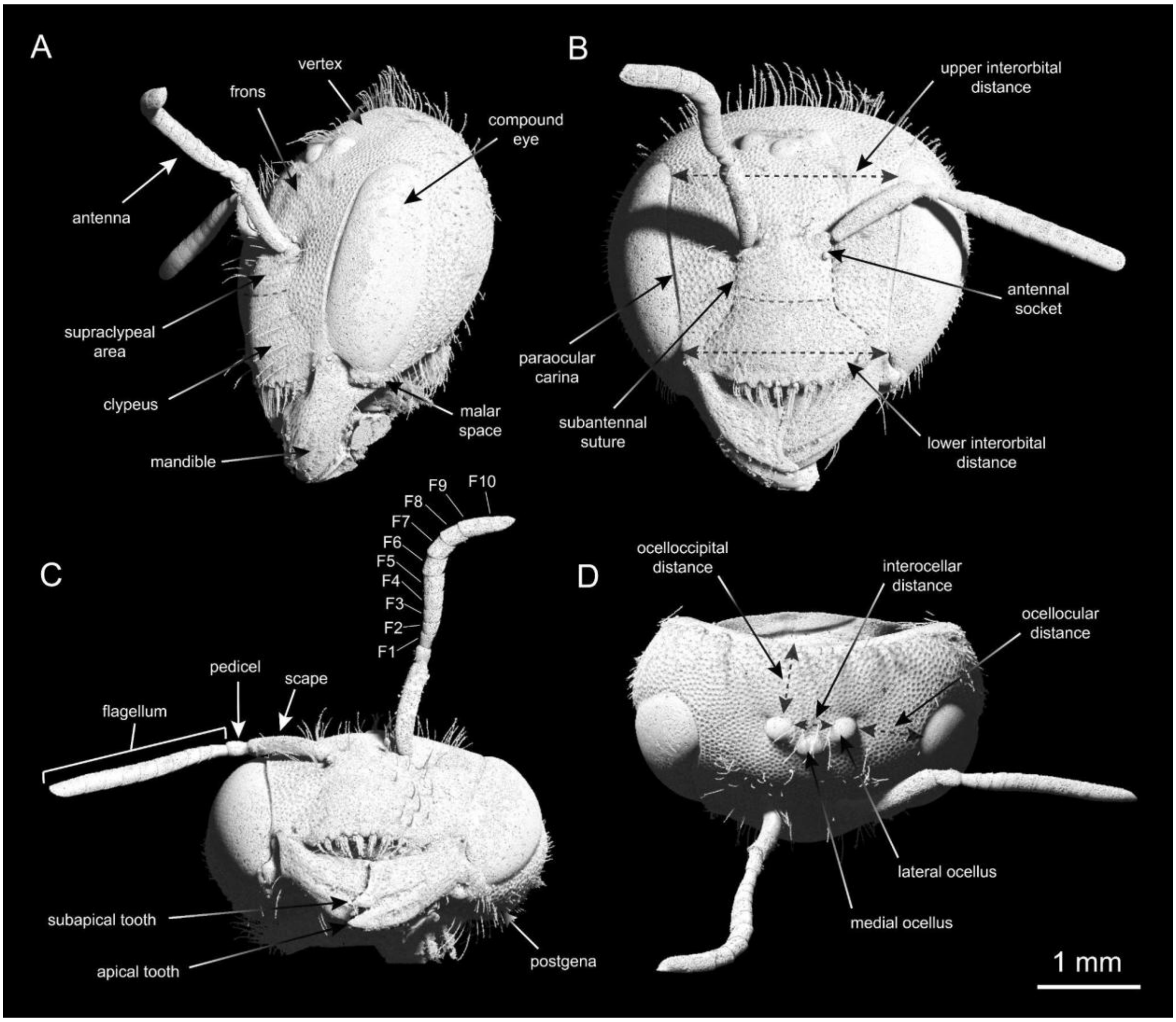

Figure S1. Head anatomy of *Protolithurgus acarophorus* nov. sp. (A) lateral. **(B)** anterior. **(C)** ventral. **(D)** dorsal. Scale bar = 1 mm.

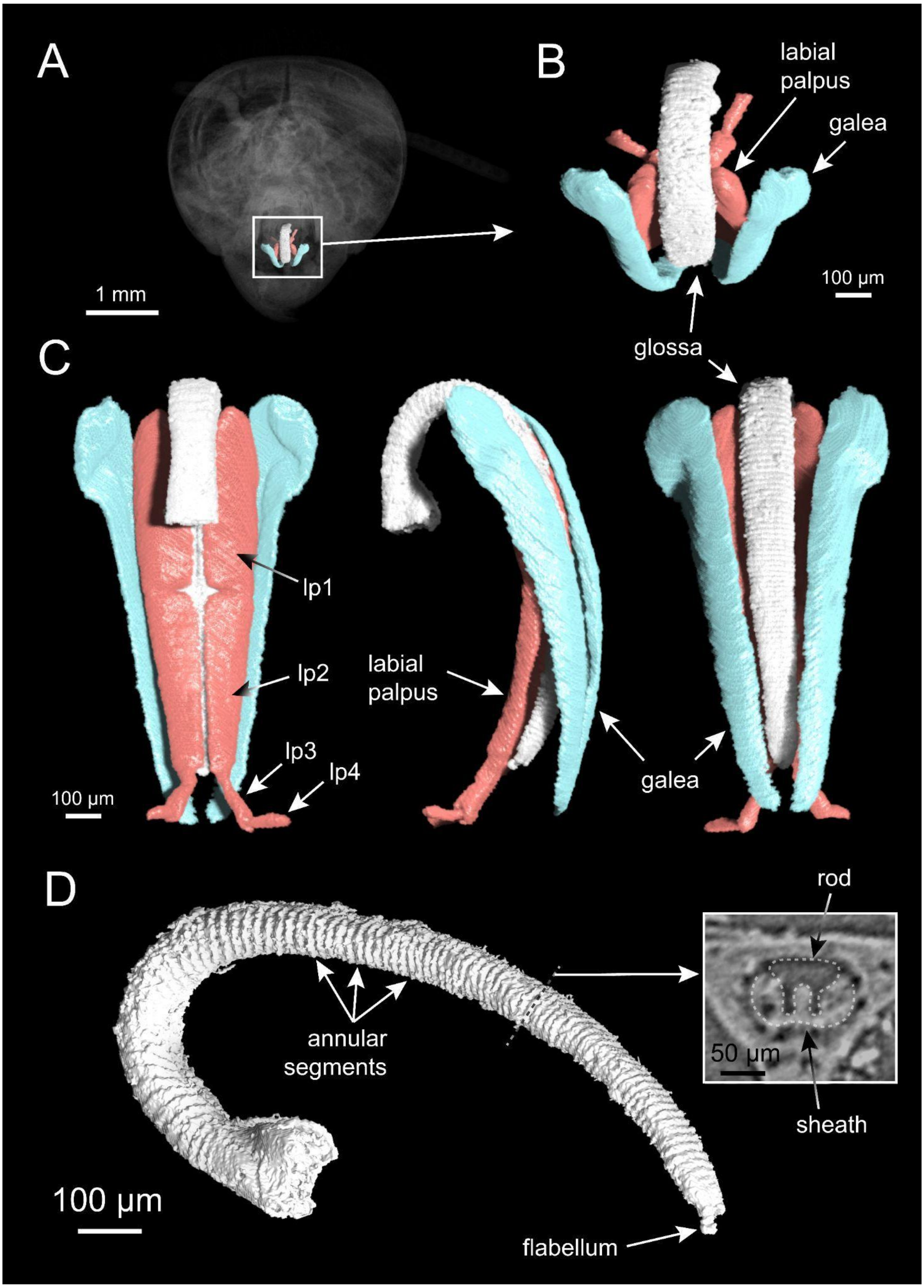

Figure S2. Identifiable mouthparts of *Protolithurgus acarophorus* nov. sp. (A) position of mouthparts within head. **(B)** dorsal mouthparts. **(C)** mouthparts in posterior, lateral, and anterior view. **(D)** close up of glossa with cross-section showing the fine morphology of the rod and sheath. Scale bar in A = 1 mm. Scale bar in C = 0.1 mm. Scale bar in D = 0.1 mm. Scale bar in D (inset) = 0.05 mm.

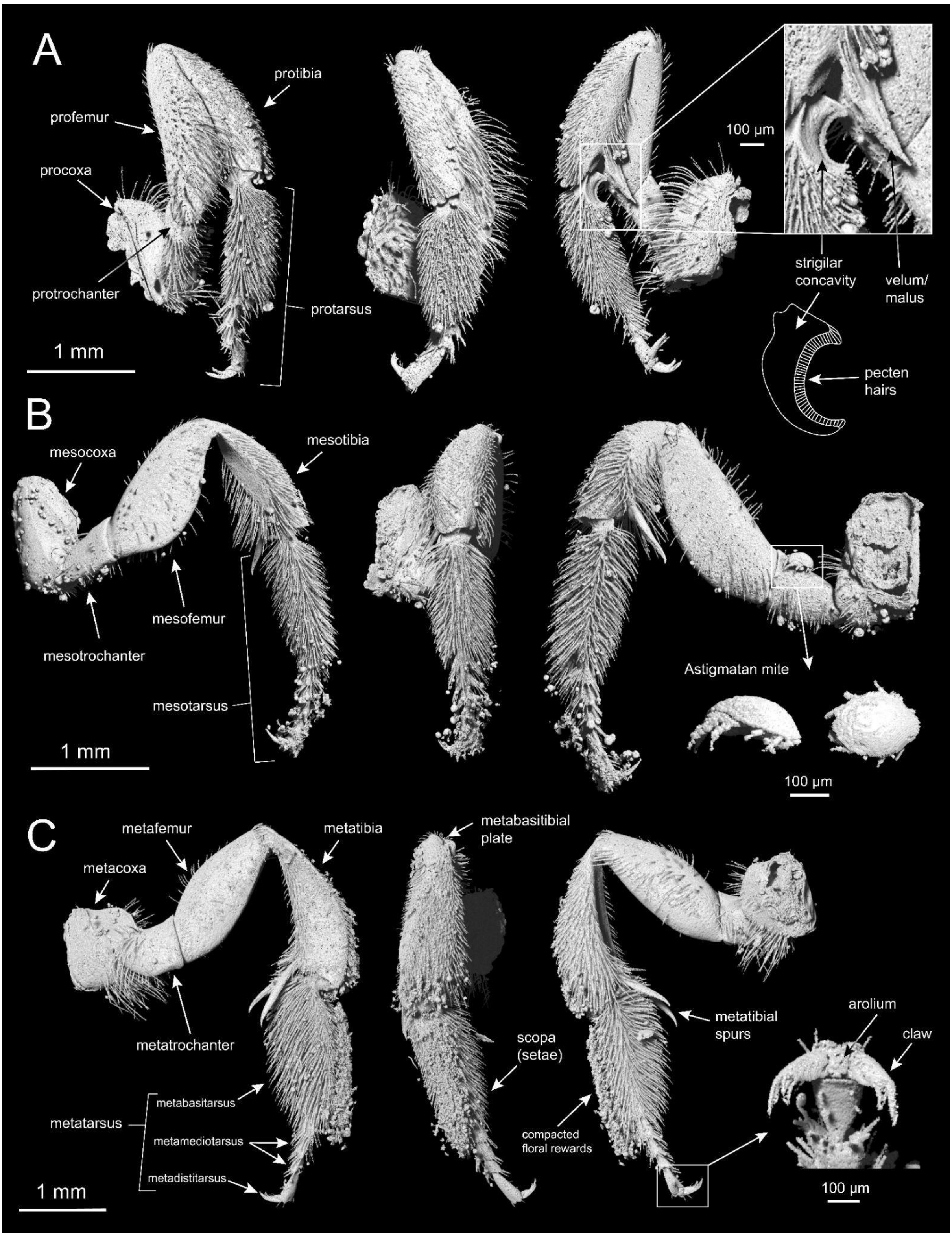

Figure S3. Leg anatomy of *Protolithurgus acarophorus* nov. sp. (A) foreleg with close-up of antennae cleaning apparatus. **(B)** midleg with close-up of Astigmatan mite on mesotrochanter. **(C)** hindleg with close-up of metatarsal claws. Note each leg is on a different scale. Main scale bars in A-C = 1 mm. Inset scale bars in A-C = 0.1 mm.

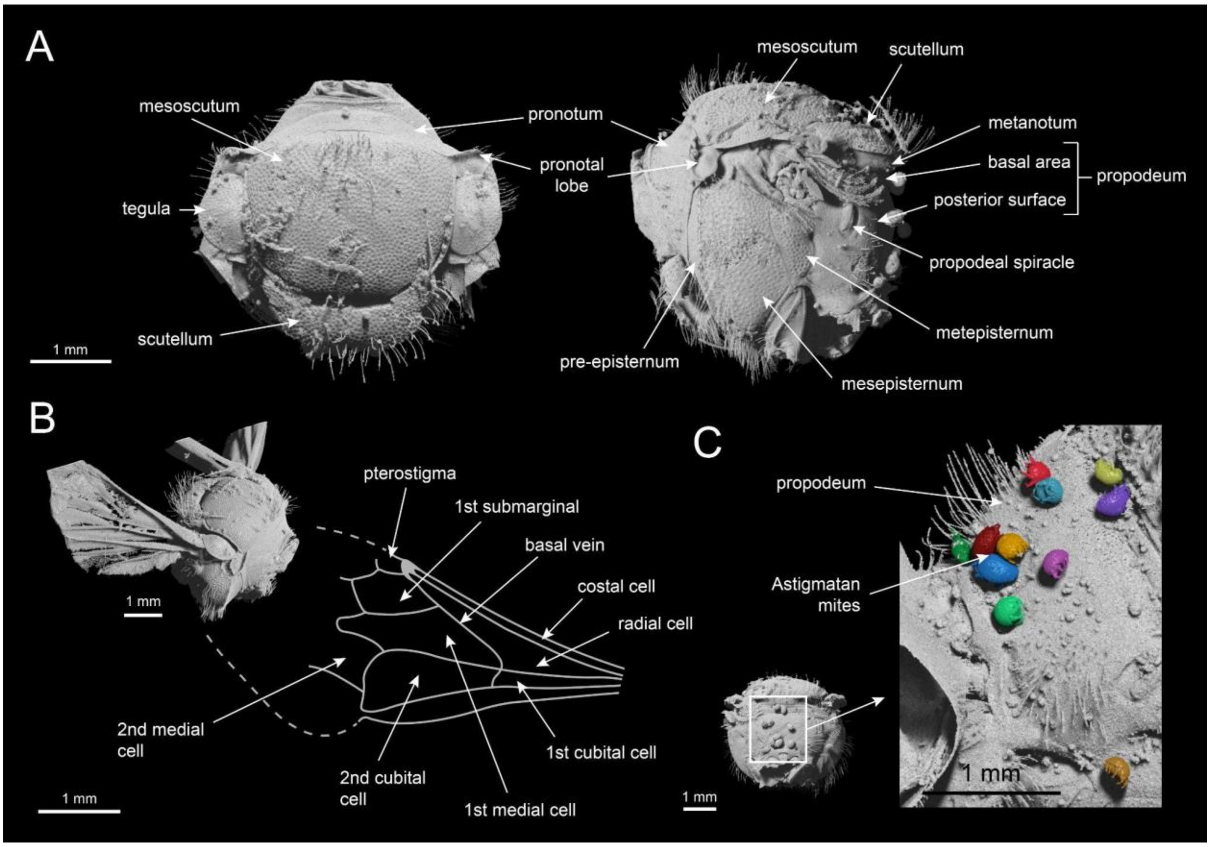

Figure S4. Mesosoma (thorax) anatomy of *Protolithurgus acarophorus* nov. sp. (A) Mesosoma in dorsal and lateral view. **(B)** Identifiable cells of the right forewing. **(C)** Astigmatan mites of the propodeum. Note that not all setae were possible to render, so refer to description for details of setae. All scale bars = 1 mm.

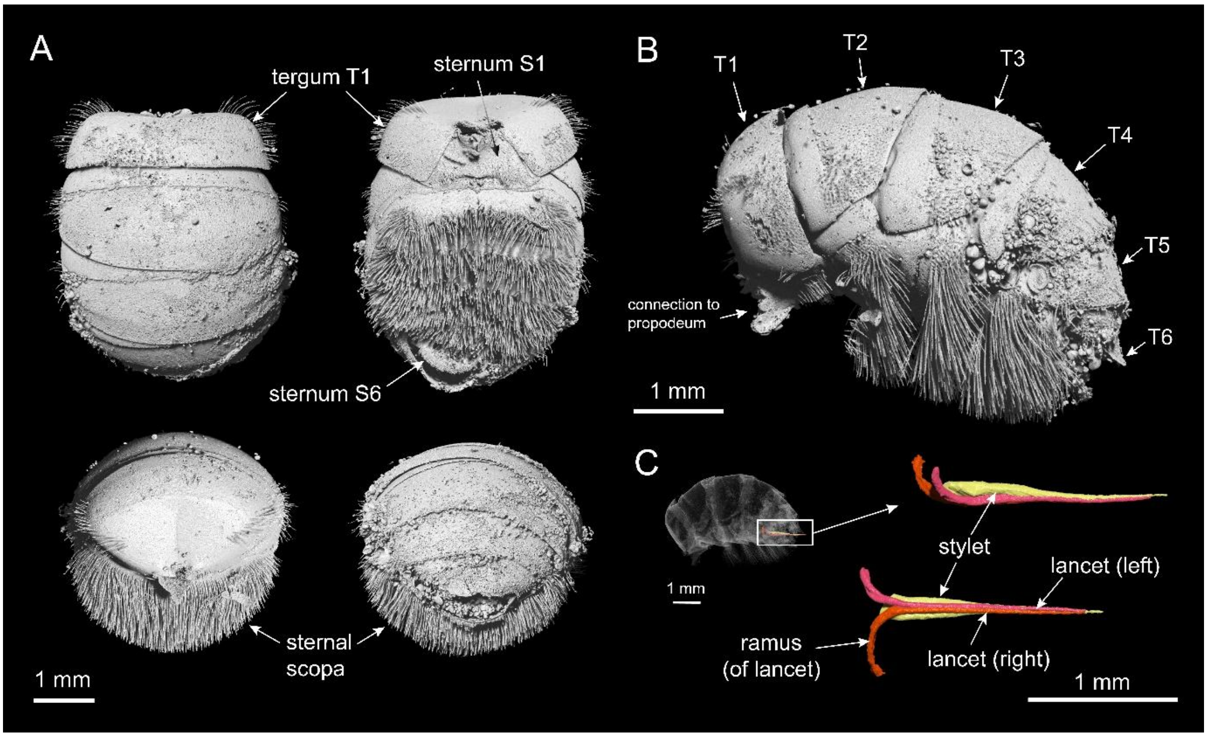

Figure S5. Metasoma (abdomen) anatomy of *Protolithurgus acarophorus* nov. sp. (A) Metasoma in dorsal (top left), ventral (top right), anterior (bottom left), and posterior (bottom right) views. **(B)** Lateral view of metasoma showing tergites. **(C)** Position and anatomy of identifiable stinger components. All scale bars = 1 mm.

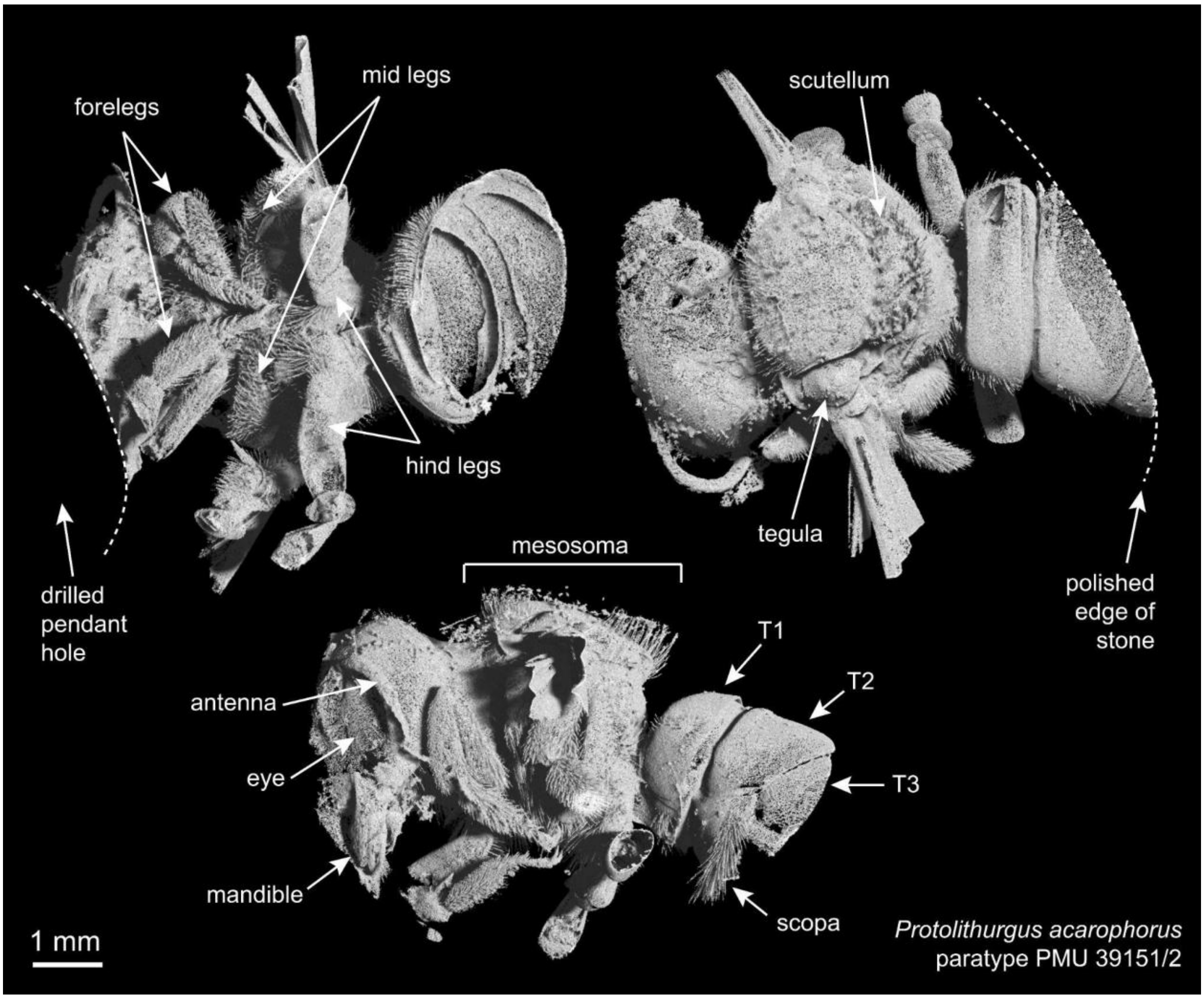

Figure S6. Habitus and general features of the paratype of *Protolithurgus acarophorus* nov. sp. (Megachilidae: Protolithurgiini). Scale bar = 1mm.

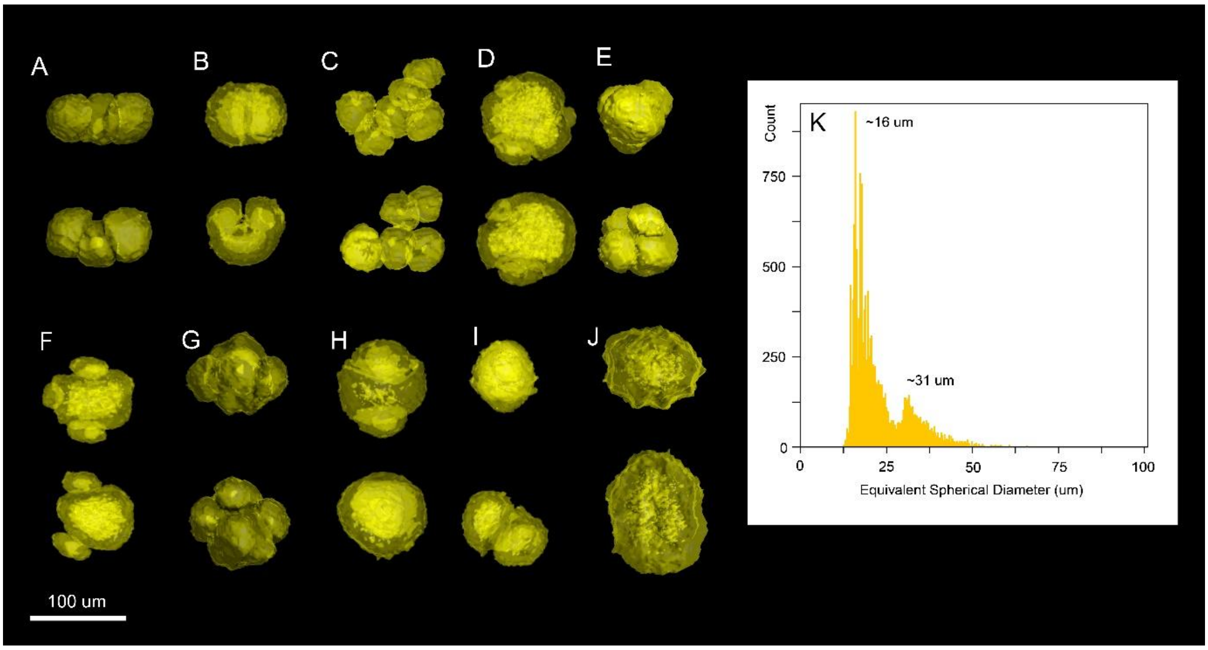

Figure S7. Large palynomorphs of distinct morphologies found on the holotype of *P. acarophorus* **(A)** Pine pollen (Pinaceae: *Pinus*); ~110 x 48 µm. **(B)** Cedar pollen (Pinaceae: *Cedrus*); ~79 x 49 µm. **(C)** Small, spherical, unidentified; ~40 x 40 µm. **(D)** Tricolpate unidentified; ~98 x 96 µm. **(E)** Possible Tetrade, unidentified; ~68 x 62 µm. **(F)** Unidentified; ~95 x 92 µm. **(G)** Unidentified; ~105 x 100 µm. **(H)** Tricolpate unidentified; ~94 x 93 µm. **(I)** Unidentified; ~94 x 60 µm. **(J)** Unidentified; ~96 x 132 µm. **(K)** Spherical diameter distribution of likely pollen grains, highlighting that most pollen grains are below the size where we can identify their morphology given our current imaging resolution. Note in panels A-J the exine (solid colors) which can be seen inside the pollen grains, most clearly in A. Scale bar = 0.1 mm.

Table S1. Mite measurements

Mite ID	Isiosoma length (µm)	Isiosoma width (µm)	Maximum leg length (µm)	Gnathosoma length (µm)
1	204	146	105	56
2	285	200	138	69
3	284	199	129	66
4	246	166	110	58
5	221	174	103	69
6	237	198	120	57
7	216	168	116	65
8	233	160	118	60
9	273	183	115	59
10	269	164	94	47
11	213	186	108	53
12	254	184	105	54
